# Towards implementation of independent calculation-based dose verification in proton therapy

**DOI:** 10.1371/journal.pone.0326525

**Published:** 2025-06-17

**Authors:** Nichakan Chatchumnan, Mananchaya Vimolnoch, Sakda Kingkaew, Puntiwa Oonsiri, Sornjarod Oonsiri

**Affiliations:** Division of Radiation Oncology, Department of Radiology, King Chulalongkorn Memorial Hospital, The Thai Red Cross Society, Bangkok, Thailand; Northwestern University Feinberg School of Medicine, UNITED STATES OF AMERICA

## Abstract

**Background:**

Proton therapy offers precision in targeting tumors while minimizing damage to surrounding healthy tissues. Independent dose calculation is a secondary dose validation tool for patient-specific quality assurance (PSQA) using Monte Carlo simulations.

**Purpose:**

To implement independent dose calculation for PSQA in proton therapy and establish confidence limits, tolerance limits, and action limits.

**Methods and Materials:**

The point dose validation of independent dose calculation was performed by ionization chamber. The dose distributions between independent dose calculation and treatment planning calculation were compared in single and multiple energy plans. Fifty clinical plans covering five regions (head and neck, breast, chest, abdomen, and pelvis) were evaluated using the gamma analysis criteria of 3%, 3 mm, and 5%, 3 mm. Confidence limits, tolerance limits, and action limits were determined.

**Results:**

The dose differences between measurement and independent dose calculation for each energy level were within 1.0%. The results showing gamma passing rates of 99.4 ± 0.6% for single energy plans and over 98.0% for multiple energy plans. The average gamma passing rates for all treatment sites was 95.9 ± 2.7% with 3%/3 mm criteria, which increased to 97.9 ± 1.8% with 5%/3 mm criteria. The confidence limits, tolerance limits, and action limits showed 90.7%, 89.1%, and 85.4% with 3%/3 mm criteria and 94.3%, 93.2%, and 91.6% for 5%/3 mm criteria, respectively.

**Conclusions:**

The independent dose calculation could potentially be implemented in PSQA. It is a secondary dose validation method for busy clinic. Particular attention should be paid to a confidence limit, tolerance limit, and action limit for reliable treatments.

## Introduction

The pencil beam scanning (PBS) technique represents a significant advancement in proton therapy, offering enhanced precision and adaptability compared to passive scatter proton therapy. This method facilitates the delivery of intensity-modulated proton therapy (IMPT), which involves finely tuned spot placement and energy modulation to achieve optimal dose distribution. Ensuring patient safety through rigorous quality assurance (QA) is essential in IMPT. Traditional measurement-based QA, where doses are verified using physical detectors [1,2], remains common but has limitations, such as time constraints and the inability to represent the actual patient geometry and heterogeneity [3].

Recent developments in independent dose calculation methods have introduced an alternative approach for dose verification using Monte Carlo (MC) simulations. This method utilizes DICOM CT datasets to account for patient-specific geometry and heterogeneities, potentially reducing the time required for the measurement procedure. Despite these advancements, there is limited research on the comparative efficacy of independent calculation-based methods versus traditional measurement-based methods in IMPT [4].

This study aims to implement independent dose calculations for patient-specific quality assurance in proton therapy. The dose distributions obtained from independent calculations were compared with measurements from 2D detectors using gamma passing rates. Additionally, confidence limits, tolerance limits, and action limits of independent calculation-based methods were determined to ensure the reliability of this approach.

## Materials and methods

The Varian ProBeam Compact spot scanning system (Varian Medical Systems, Palo Alto, California) was used, capable of delivering proton beams within the energy range of 70–220 MeV. Proton treatment plans were created using the proton convolution superposition (PCS) algorithm in the Varian Eclipse Treatment Planning System (TPS), version 16.1. Dose distributions from the TPS were compared with independent dose calculations performed using the myQA iON software version 2.1 (IBA Dosimetry, Schwarzenbruck, Germany) with MCsquare algorithm [5].

In this study, we conducted validation through three main processes, as follows:

### 2.1 Point dose validation

Point dose measurements were conducted for proton energies of 70, 100, 130, 160, 190, and 200 MeV. A PTW30013 ionization chamber (PTW Freiburg, Germany) with 0.6 cm3 sensitive volume, 6.2 mm inner diameter and 24 mm length was placed in a Virtual Water™ phantom (Standard Imaging Inc., Middleton, WI) at 2 cm depth, 2 Gy in a 10 × 10 cm² field size, 100 MU per spot, total spots of 1681 spots per layer according to IAEA TRS 398 and AAPM TG185 [6,7]. The accuracy of myQA iON calculations was validated against these measurements in term of percentage dose difference.

### 2.2 Simple plan validation

**Fig 1:**
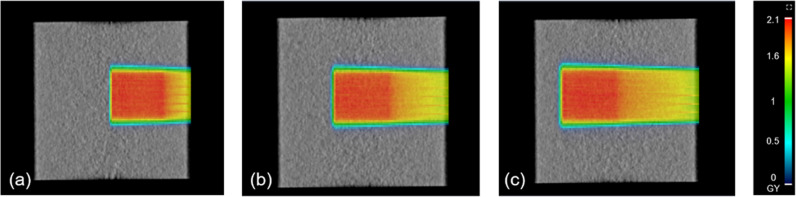
Calculation of multiple energy plans with 10 × 10 × 10 cm3 cubic size by myQA iON at different depths: (a) 10 cm depth, (b) 15 cm depth, and (c) 20 cm depth.

For pencil beam scanning, both single and multiple energy plans were assessed. Single energy plans were generated with a 10 × 10 cm² field size, 2Gy normalized at 2 cm depth, for each energy (70, 100, 130, 160, 190, and 200 MeV), with gamma analysis applied using 3%/3 mm criteria [4]. Multiple energy plans were generated in a cubic shape by vary size of field size included cubic targets of various sizes (3 × 3 × 3, 5 × 5 × 5, 7 × 7 × 7, 10 × 10 × 10, and 15 × 15 × 15 cm^3^) positioned at depths of 10, 15, and 20 cm. Example dose distributions for a 10 × 10 × 10 cm^3^ cubic size at different depths are shown in Fig 1. The treatment plans with and without range shifter of 2,3 and5 cm thickness were validated in 10 × 10 × 10 cm^3^ in 15 cm depth with gamma analysis applied using 3%/3 mm criteria.

### 2.3 Clinical validation

Patient-specific quality assurance (PSQA) was conducted for a total of an anonymous fifty treatment plans across five anatomical sites: head and neck (H&N), breast, chest, abdomen, and pelvis from February to October in 2024. The requirement for informed consent was waived by the IRB. Each plan was generated using the TPS as a verification plan, with dose calculations performed for a solid water phantom at an appropriate depth to compare with measurement. Measurements were performed using the PTW OCTAVIUS Detector 1500XDR array (PTW-Freiburg, Germany) at isocenter. The measurements were performed following our previous study with 3%/2 mm gamma criteria [2].

Independent dose calculation-based, all patient plans consisting of the DICOM CT datasets, RT plan, RT structure, and RT dose, were exported to myQA iON for recomputation on the patients CT geometry using the gamma analysis criteria of 3%, 3 mm, and 5%, 3 mm [4]. A confidence limits analysis was performed on myQA iON recomputation data to evaluate the reliability of local facility data in terms of the percentage of gamma passing based on a formula from AAPM TG‐119 [3] using the following equation,


$$\begin{array}{c} \text{Confidence\textbackslash{} limit\textbackslash{} }=\;|\text{mean}|\;+1.96\text{ SD} \end{array}$$ (1)

Tolerance and action limits were determined according to the equations provided in AAPM TG-218 [8]. Specifically, tolerance limits were calculated using the I-chart statistical tool, which includes the center line, upper control limit, and lower control limit. These limits were computed using the following equations:


$$\begin{array}{c} \text{Center\textbackslash{} line}=\;\frac{1}{n}\sum_{1}^{n}x \end{array}$$ (2)

$$\begin{array}{c} \text{Upper\textbackslash{} control\textbackslash{} line }=\text{\textbackslash{} center\textbackslash{} line}+2.660\cdot \;\overset{―}{\text{mR}} \end{array}$$ (3)

$$\begin{array}{c} \text{Lower\textbackslash{} control\textbackslash{} line}=\text{ \textbackslash{} center\textbackslash{} line}+2.660\cdot \;\overset{―}{\text{mR}} \end{array}$$ (4)

where x is an individual calculation, n is the total number of calculations, and $\overset{―}{mR}=\;\frac{1}{n-1}\sum_{i=2}^{n}\left|x_{i}-\;x_{i-1}\right|\;$ is the moving range.

Action limits are calculated using the following equation,


$$\Delta A=\;\beta \sqrt{\sigma^{2}+{\left(\overset{―}{x}-T\right)}^{2}}$$ (5)

Where ∆A is the difference between the upper and lower action limits, T is the target value (100), $\sigma^{2}$ is the process variance, $\overset{―}{x}\;$ is the process mean, and β is a combination of two factors, which suggests that β = 6.0 is an appropriate value to use.

**For statistical analysis, variance analysis (ANOVA) was used to compare differences between regional sites, and a paired t-test was employed to assess differences between the measurement data and the independent software data. A significance level of p < 0.05 was considered for all statistical tests.**

## Results

### 3.1 Point dose validation

Table 1 summarizes the percentage of dose differences between measurement and independent dose calculation for each energy level. Overall, differences were within 1.0%, with a minimum difference of 0.48% at 130 MeV and a maximum of 0.96% at 160 MeV.

**Table 1:** Percentage dose differences comparison between measurement and independent dose calculation.

Energy (MeV)	Dose (Gy)		% Difference
	Measurement	Independent dose calculation	
70	2.00 ± 0.03	2.02	0.78
100	2.01 ± 0.00	2.03	0.75
130	1.98 ± 0.03	1.99	0.48
160	1.99 ± 0.00	2.01	0.96
190	2.02 ± 0.02	2.03	0.68
200	2.00 ± 0.02	2.01	0.58

### 3.2 Simple plan validation

For single energy plans, the average gamma passing rates were 99.4 ± 0.6%, with a minimum of 98.0% at 70 MeV (Table 2). Multiple energy plans showed gamma passing rates exceeding 98.0% for most cubic sizes and depths, except for smaller cubic sizes of 3 × 3 × 3 and 5 × 5 × 5 cm^3^ at 10 cm depths (Table 3).

**Table 2:** Gamma passing rates of single energy plans.

Energy (MeV)	Gamma passing rates (%)
70	98.0
100	99.7
130	99.7
160	99.8
190	99.3
200	99.3
**Average**	**99.4 ± 0.6**

**Table 3:** Gamma passing rates of multiple energy plans for various cubic sizes and depths.

Depth (cm)	Gamma passing rates (%)				
	3 × 3 × 3 cm^3^	5 × 5 × 5 cm^3^	7 × 7 × 7 cm^3^	10 × 10 × 10 cm^3^	15 × 15 × 15 cm^3^
10	95.7	97.8	98.5	99.3	99.8
15	100.0	100.0	99.7	99.7	99.6
20	99.4	98.9	99.5	99.1	99.1

Table 4 shown a gamma passing rate of 100% in plan without range shifter and slightly decrease in plans with range shifter 2 cm, 3 cm and 5 cm at 96.8%, 97.4% and 97.2% with 3%/3 mm gamma criteria.

**Table 4:** Gamma passing rates of with and without range shifter plans in 10 × 10 × 10 cm^3^ at 15 cm depth.

Range shifter	Gamma passing rates (%)
Without	100
2 cm	96.8
3 cm	97.4
5 cm	97.2

### 3.3 Clinical validation

The measurement-based shown an average gamma passing rate of 99.2 ± 1.6% using the 3%/2 mm criteria across all treatment sites, as detailed in Table 5. The minimum gamma passing rates of 98.8 ± 1.9% was observed in abdomen region, while showed maximum of 99.6 ± 0.7% in breast region.

**Table 5:** Gamma passing rates comparison between measurement and myQA iON for various clinical treatment sites.

Treatment sites	Gamma passing rates (%)			*p-value*	
	Measurement	Independent dose calculation			
	3%/2 mm	3%/3 mm	5%/3 mm	Measurement Vs 3%/3 mm	Measurement Vs 5%/3 mm
H&N	99.4 ± 1.2	95.8 ± 1.9	98.0 ± 1.3	<0.05	<0.05
Breast	99.6 ± 0.7	95.8 ± 2.6	98.4 ± 1.2	<0.05	0.36
Chest	99.0 ± 1.9	94.6 ± 2.5	97.0 ± 1.9	<0.05	<0.05
Abdomen	98.8 ± 1.9	95.2 ± 2.4	96.9 ± 1.8	<0.05	<0.05
Pelvis	99.4 ± 1.2	98.2 ± 2.4	99.0 ± 1.7	0.10	0.22
**Average**	**99.2 ± 1.6**	**95.9 ± 2.7**	**97.9 ± 1.8**	**–**	**–**

**Fig 2:**
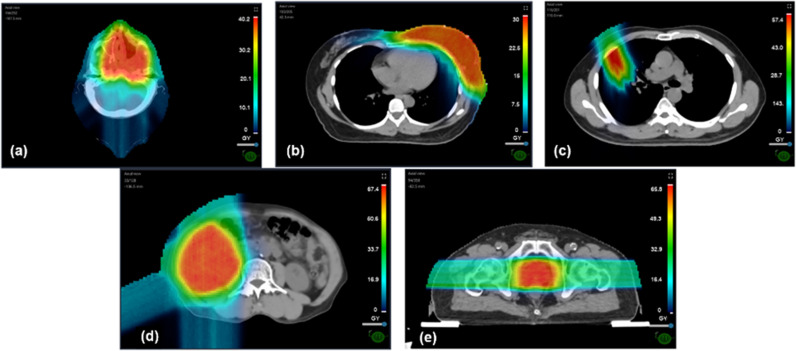
Calculation of dose distribution by independent dose calculation in different treatment sites: (a) Head and neck, (b) Breast, (c) Chest, (d) Abdomen, and (e) Pelvis.

Independent dose calculation-based, the average gamma passing rates for all treatment sites presented results of 95.9 ± 2.7% with 3%/3 mm criteria, which increased to 97.9 ± 1.8% when applying the 5%/3 mm criteria. An example of independent dose calculations is shown in Fig 2. The average gamma passing rates for each treatment site and gamma criteria are shown in Table 5. With 3%/3 mm criteria, the minimum gamma passing rate of 94.6 ± 2.5% was shown in the chest region and a maximum of 98.2 ± 2.4% in the pelvis. With the 5%/3 mm criteria, the abdomen region showed the minimum gamma passing rates of 96.9 ± 1.8%, while the maximum of 99.0 ± 1.7% was observed in the pelvis region.

The results indicate the significant differences between independent dose calculation and measurement (p < 0.05) in both 3%/3 mm and 5%/3 mm criteria. However, there are no significant differences in the breast region with the 5%/3 mm criterion (p = 0.36) and in the pelvis region for both criteria (p = 0.10 and p = 0.22).

The confidence limits, tolerance limits, and action limits were performed with 3%/3 mm and 5%/3 mm criteria, as presented in Table 6. With 3%/3 mm criteria, confidence limits, tolerance limits, and action limits showed 90.7%, 89.1%, and 85.4%, respectively. For 5%/3 mm criteria, confidence limits, tolerance limits, and action limits showed 94.3%, 93.2%, and 91.6%, respectively.

**Table 6:** Confidence limits, tolerance limits, and action limits of independent dose calculation with difference gamma criteria.

Treatment sites	Independent dose calculation					
	Confidence limits (%)		Tolerance limits (%)		Action limits (%)	
	3%/3 mm	5%/3 mm	3%/3 mm	5%/3 mm	3%/3 mm	5%/3 mm
H&N	92.0	95.6	90.4	94.1	86.2	93.0
Breast	90.7	96.0	88.3	94.3	85.2	94.0
Chest	89.6	93.3	87.2	91.9	82.0	89.3
Abdomen	90.6	93.4	89.0	90.7	84.0	89.3
Pelvis	93.4	95.7	91.4	95.2	90.9	94.1
**All regions**	**90.7**	**94.3**	**89.1**	**93.2**	**85.4**	**91.6**

## Discussion

The independent dose calculation performed accuracy calculations for all energy types, including single and multiple energy plans. The independent dose calculation also showed gamma passing rates of more than 95% with 3%/3 mm and more than 97% with 5%/3 mm criteria in clinical plans.

For point dose validation, our study observed a dose difference between measurement and independent dose calculation of 0.7 ± 1.5%. Comparable to Huang et al. [9] and Fracchiolla et al. [10] reported, the mean point-to-point dose differences between the Bragg Peak curves below 0.5%. However, our results were within 1.0% agree with the criteria of acceptability by IAEA Technical Reports Series No.430, which applied for a square field at central rays in homogeneous phantoms [11].

For multiple energy plans, the results showed that gamma passing rates generally improve with larger cubic sizes and greater depths corresponding with Coholis et al. study [12]. Our study obtained the average gamma passing rate across various cubic sizes of 99.1 ± 1.1% agree with Guterres Marmitt et al. [13], which achieved a 99.0 ± 0.5% gamma passing rate from 30 SOBPs covering energy ranges from 70 to 225 MeV with vary cubic sizes of 20–40 mm.

For clinical validation, the measurement-based study demonstrated an average gamma passing rate of 99.2 ± 1.6% using the 3%/2 mm criteria, according to our previous study [2] which obtained the confidence limits of 95.7%. The AAPM TG-219 report sets action limits of 88–90% with the 3%/2 mm gamma criteria for composite irradiations applied to independent calculation-based systems for photons [4]. The AAPM TG-219 report acknowledges limitations in the algorithm for independent verification and recommends using a 3 mm radius criterion instead to improve this limit. The AAPM TG-219 also mentioned for a composite plan, a dose difference of 3% is suggested for homogeneous and higher to 5% for heterogeneous [4]. Labagnoy YJM et al. [14] also reported 5%/3 mm criteria for independent dose calculation in photon beams. However, the specific criteria for independent calculation-based verification to apply with a proton have not defined.

The results demonstrate the effectiveness of independent dose calculation. With the 3%/3 mm criteria, our study achieved an average gamma passing rate of 95.9 ± 1.2% in 50 treatment plans. The lowest gamma passing rate observed in the chest region and highest in the pelvis region. Comparable to Dreindl et al. [15] reported a mean gamma passing rate of 98.8 ± 1.3% for 131 clinical treatment plans, with a gamma passing rate of 99.3% in the prostate region. They also noted significant deviations, reaching −11.1%, in lung tissue when using independent dose calculations. This indicates the challenge of the dose calculation in the lung region from the independent dose calculation. Furthermore, independent dose calculation is an available option for patient-specific quality assurance within the adaptive treatment workflow [16].

This study indicates that the data transfer from the TPS to the OIS or myQA iON is accurate based on our results. However, limitations exist in the data transfer process from the OIS to the treatment machine due to the absence of third-party conversion into a log file format. Future studies will incorporate log files or DICOM treatment records into patient-specific quality assurance workflows.

In conclusion, the independent dose calculation could potentially be implemented in PSQA for most treatment sites. The myQA iON is a useful secondary dose calculation but is not a replacement for IMPT QA measurements. It is a secondary dose calculation method for busy clinic. However, careful attention should be given when applying in inhomogeneities and high-density materials. Furthermore, tolerance and action limits should be established based on clinical data at each center to ensure reliable and accurate treatments.
